# Calcium-sensing receptors promoted Homer1 expression and osteogenic differentiation in bone marrow mesenchymal stem cells

**DOI:** 10.1515/biol-2022-1059

**Published:** 2025-03-07

**Authors:** Kainan Liu, Tianjie Xu, Jiaxin Fan, Yueyuan Li, Xiaoling Guo, Hui Zhang, Qian Wang

**Affiliations:** School of Basic Medical Sciences, North China University of Science and Technology, Bohai Road, Caofeidian District, Tangshan, Hebei, 063210, China; Department of Basic Medicine, Xingtai Medical College, Xingtai, Hebei, 054000, China; Department of Joint Surgery, The Second Hospital of Tangshan, Tangshan, Hebei, 063000, China

**Keywords:** bone marrow mesenchymal stem cells, calcium-sensing receptor, osteogenic differentiation.

## Abstract

Homer1 interacts with calcium-sensing receptors (CaSRs) in osteoblasts (OBs), with both CaSR and Homer1 playing essential roles in AKT phosphorylation. This study investigated the impact of CaSR on Homer1 expression during the differentiation of rat bone marrow mesenchymal stem cells (BMSCs) at morphological, imaging, and molecular levels, both *in vivo* and *in vitro*. A post-oophorectomy osteoporosis model was established in Sprague-Dawley rats, validated through micro-computed tomography, hematoxylin-eosin staining, and biomechanical testing to assess *in vivo* changes in CaSR expression. BMSCs were isolated from 3 week-old SD rats for *in vitro* OB differentiation studies, wherein osteogenic differentiation was induced alongside changes in CaSR expression. Morphological alterations were examined using transmission electron microscopy and immunofluorescence staining. Furthermore, the protein and mRNA levels of OB-specific genes were quantified by Western blot and reverse transcription quantitative real-time polymerase chain reaction, with Homer1-related proteins also assessed. Results showed a reduction in CaSR and Homer1 expression in the ovariectomized group. In cellular studies, CaSR activation upregulated AKT, Homer1, and osteogenic markers, promoting cell differentiation. In conclusion, CaSR enhances OB differentiation, likely via Homer1-mediated regulation of AKT signaling, suggesting CaSR as a potential therapeutic target for osteoporosis.

## 1 Introduction

Osteoporosis (OP) is a systemic bone disorder characterized by reduced bone mass, impaired bone microstructure, increased bone fragility, and an elevated risk of fractures [1]. It arises from an imbalance between bone resorption by osteoclasts (OCs) and bone formation by osteoblasts (OBs) [2]. Bone marrow-derived mesenchymal stem cells (BMSCs) have the potential to differentiate into multiple cell types, maintaining a critical balance essential for normal bone metabolism. This balance is disrupted in the OP state [3]. A central mechanism underlying OP is the impaired differentiation of BMSCs into OBs [4]. Research has demonstrated that both the number of BMSCs and their osteogenic differentiation capacity are altered in patients with OP, resulting in impaired bone formation [5]. Consequently, exploring the molecular mechanisms regulating BMSC differentiation into OBs is essential for understanding OP pathogenesis and developing effective therapeutic strategies for its prevention and treatment.

The calcium-sensing receptor (CaSR), a G-protein-coupled receptor (GPCR), plays a pivotal role in calcium homeostasis [6]. It regulates Ca^2+^ and other metal ions while promoting cell proliferation and differentiation [7]. Activation of CaSR can modulate the release of transforming growth factor-β (TGF-β), which in turn stimulates OB proliferation, differentiation, and mineralization via the TGF-β type I/II receptor (TβRI/II)-Smad 2/3 signaling pathway. Studies have shown that CaSR activation triggers various signaling cascades in osteocytes, promoting OB proliferation and differentiation, inhibiting OC activity, and thereby exerting therapeutic effects in OP treatment [8,9]. Moreover, the ERK1/2-MAPK, Wnt/β-catenin, and Akt signaling pathways rely on CaSR to regulate OB function [9].

Homer proteins, initially discovered in the nervous system [10,11], function as scaffold proteins. The primary isoforms include Homer1, Homer2, and Homer3. Homer1 is known to regulate calcium channels, act as a scaffold protein, and modulate the endoplasmic reticulum calcium release channel [12]. Additionally, Homer1 plays a role in mediating AKT phosphorylation. Activation of CaSR induces AKT Ser 473 phosphorylation and nuclear β-catenin translocation in OBs, with Homer proteins promoting AKT phosphorylation at the cell membrane [13]. The involvement of CaSR and Homer1 in AKT Ser 473 and GSK 3β-S9 phosphorylation has been demonstrated in MG 63 osteosarcoma cells. However, the exact role of Homer1 in osteogenic differentiation and OP remains unclear.

This study hypothesizes that Homer1 plays a role in OP pathogenesis, with CaSR promoting OB proliferation and differentiation by modulating Homer1, thereby exerting a therapeutic effect on OP. To test this hypothesis, a bilateral ovariectomized rat model of OP was established, as ovarian ligation-induced models are commonly used to study OP pathology and explore potential therapeutic strategies [14]. Additionally, rat BMSCs were used to investigate the mechanism by which CaSR influences osteogenic differentiation. Specifically, this study aimed to determine whether Homer1 expression was altered in the rat model of postmenopausal OP and whether Homer1 participates in osteogenic differentiation through CaSR signaling. These findings may provide insights for the development and optimization of novel diagnostic and therapeutic approaches for OP.

## 2 Materials & methods

### 2.1 Animals

All animal procedures were performed in compliance with the Guidelines for the Care and Use of Laboratory Animals set by the National Institutes of Health. The animals were provided by the Animal Center of North China University of Science and Technology (License No.: SYXK(Ji)2020-007). Eight week-old female rats (180–200 g) and 3 week-old male SD rats were selected for the study. The animals were housed under standard laboratory conditions.


**Ethical approval:** The research related to animal use has been complied with all the relevant national regulations and institutional policies for the care and use of animals, and has been approved by the Experimental Animal Ethics Committee of North China University of Science and Technology (Approval No.: 2023-SY-015).

### 2.2 Rat model of ovariectomized osteoporosis (OVX)

**Figure 1:**
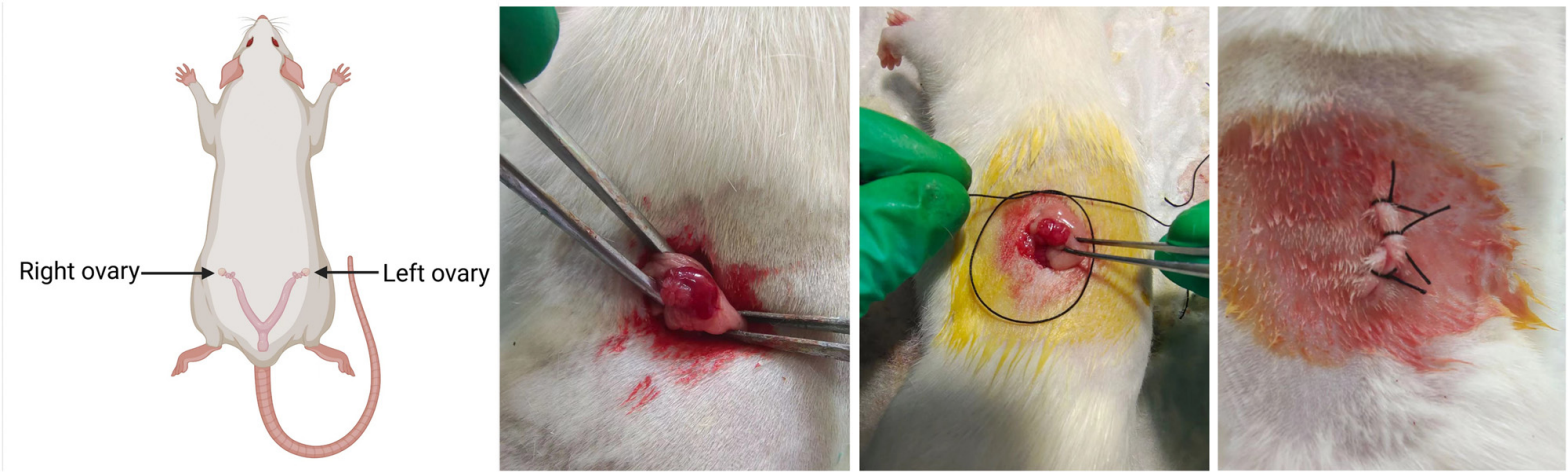
Diagram of the OVX rat model. Created with BioRender.com.

Female SD rats were placed in a barrier environment. After a 1 week acclimation period, the rats were randomly assigned to two groups (*n* = 5): the sham group and the ovariectomized (OVX) group. Prior to surgery, the rats were fasted for 12 h and anesthetized with 60 mg/kg sodium pentobarbital. The rats were fixed in a lateral position, and a 1–2 cm incision was made on the lateral abdomen. The skin was incised with scissors, and the fascia and muscle were separated bluntly. White adipose tissue was exposed, and the ovaries, which appeared as pink, cauliflower-shaped structures, were visible upon removal of the fat folds. The ovariectomy model was established by ligating the fallopian tubes and removing the ovarian tissue (Specific operations shown schematically in Figure 1. Created with BioRender.com.) [15]. In the Sham group, adipose tissue of equivalent weight to the ovaries was used. The adipose tissue was then returned to the abdominal cavity, and an appropriate amount of penicillin was applied to prevent infection. The surgical sites were closed in sequence: muscle, fascia, and skin. Postoperatively, the rats were monitored, and after recovery, they were fed routinely with *ad libitum* access to food and water.

Eight weeks post-surgery, all female rats were anesthetized and sacrificed via cervical dislocation. Tibias and femurs were carefully isolated, fixed in 4% paraformaldehyde, or frozen for subsequent experiments. The fixed tibias were decalcified in 4% ethylenediaminetetraacetic acid disodium salt (EDTA disodium) (Coolaber, China, CE31192504) for 3 months. After decalcification, the samples were dehydrated in a graded series of ethanol. Following clearing in xylene, the samples were embedded in paraffin and sectioned into 6 μm thick slices.

### 2.3 Micro-computed tomography (Micro-CT) determination

Micro-CT (PINGSENG Healthcare Inc., China) was used to scan the proximal femur in both sagittal and horizontal planes.

The scanned data were analyzed to calculate key bone parameters, including bone mineral density (BMD, mgHA/cm^3^), which correlates positively with OP and bone strength, and bone volume/tissue volume (BV/TV, %), which correlates positively with load-bearing capacity. Additional parameters assessed included the structural model index (SMI), which is negatively correlated with trabecular resistance to compression; trabecular number (Tb.N, mm^−1^); trabecular thickness (Tb.Th, %), negatively correlated with OP, fractures, and degeneration; and trabecular separation (Tb.Sp, μm), which is inversely related to bone quality.

### 2.4 Biomechanical properties

A three-point bending test was performed on the tibia using a universal electronic testing machine (Shimadzu, Japan) to evaluate the biomechanical properties of the rats’ tibiae. The tibia samples were carefully positioned on a low support, with an 8 mm span between the two fixed loading points. A force of 2 N was applied to the tibia at a constant displacement rate of 2 mm/min until fracture occurred. The primary parameters measured during the test included maximum load, flexural stiffness, and elastic modulus.

### 2.5 Hematoxylin and eosin (H&E) staining

H&E staining was performed according to standard protocols [16]. Briefly, paraffin sections were deparaffinized in xylene and rehydrated through a graded ethanol series. The sections were stained with hematoxylin for 10 minutes, followed by thorough washing with distilled water. Differentiation was carried out using 1% hydrochloric acid alcohol for 3 s, followed by rinsing. The sections were then stained with eosin for 1 min and rinsed again. Finally, the sections were sealed and observed under a microscope. Digital images were captured using a scanning system (Olympus, Japan, CX31).

### 2.6 Immunohistochemistry (IHC)

For immunohistochemical analysis, the paraffin sections were deparaffinized using xylene and rehydrated through graded ethanol. Antigen retrieval was performed using 0.1% pancreatic enzyme at room temperature for 15 min. The sections were washed with phosphate-buffered saline (PBS) and treated with an endogenous peroxidase blocker (Zhongshan Jinqiao, Beijing, China, PV-6001). Following this, the sections were incubated overnight at 4°C with a primary antibody diluted 1:200. Then, the sections were incubated with an enzyme-conjugated goat anti-rabbit IgG polymer at room temperature for 1 h. Color development was achieved using 3,3′-diaminobenzidine (DAB), and the sections were counterstained, sealed, and observed under a microscope. Digital images were captured using a microscope scanning system. The primary antibodies used included CaSR (Abclonal, China, A1426), collagen type I (Col I; Abclonal, China, A1352), and Homer1 (Affinity, USA, DF12280).

### 2.7 Cell culture and identification

Primary BMSCs were isolated from male SD rats through standard procedures [17]. Briefly, rats were anesthetized and euthanized by cervical dislocation. The tibia and fibula were harvested under sterile conditions, with surrounding subcutaneous tissue and muscle removed. Both ends of the bones were excised, and the bone marrow cavity was flushed with α-modified Eagle’s medium (α-MEM, Eallbio, China, 03.18001A) to collect the bone marrow. The isolated cells were dissociated by pipetting and transferred to culture flasks, and a single-cell suspension was prepared. Cells were cultured in α-MEM supplemented with 10% fetal bovine serum (FBS) and 1% penicillin–streptomycin (Eallbio, China) and maintained in a humidified incubator at 37°C with 5% CO₂. Upon reaching 80% confluence, cells were detached using 0.25% trypsin and subcultured. Passage 3 (P3) cells were utilized for subsequent experiments. Characterization of third-generation BMSCs was performed via flow cytometry using antibodies against CD90 (positive marker) and CD45 (negative marker) (BD Pharmingen, China).

### 2.8 Cell viability assay

For cell proliferation assays, BMSCs were seeded at 2 × 10^3^ cells/well in 96-well plates and cultured in basal medium for 24 h. The cells were then incubated with varying concentrations of GdCl_3_ (Adams, China, P1616025) (200, 250, 300, 350, and 400 μg/mL) or NPS2390 (Cayman, USA, 11989) (5, 10, 15, and 20 μg/mL) for 5 days. Every one day, 10 μL of CCK-8 solution was added to each well and incubated for 1 h. Absorbance at 450 nm was measured using a microplate reader (Thermo Scientific, China, 117123001).

### 2.9 Cell treatment

For osteogenic differentiation, the third-generation BMSCs were induced with GdCl_3_ to activate CaSR and NPS2390 to inhibit CaSR expression. The cells were grouped into four conditions: undifferentiated (Undiff), differentiated (Diff), GdCl_3_, and NPS. In the Undiff group, BMSCs were cultured in a complete medium (α-MEM + 10% FBS). The Diff group received osteogenic induction medium (complete medium + 10 mmol/L β-sodium glycerophosphate (Alfa Aesar, USA, 41099500), 50 mg/L ascorbic acid (TCI, China, QGNMA-SG), 10^−8^ mol/L dexamethasone (TCI, China, AAVYJ-BC)). The GdCl_3_ group was treated with 300 μmol/L GdCl_3_ in the osteogenic induction medium, while the NPS group was treated with 10 μmol/L NPS2390 in the osteogenic induction medium.

### 2.10 Transmission electron microscopy (TEM)

TEM (Hitachi High-Technologies Corporation, Japan, HITACHI HT7800) was employed to visualize autophagy. On day 14, the cells were scraped, aggregated, and centrifuged to form pellets. The pellets were fixed in an electron microscopy fixation solution (Servicebio, China, G1102) at 4°C, rinsed, and then treated with 1% osmium tetroxide until blackened. Following fixation, the samples underwent dehydration, infiltration, and embedding, after which resin blocks were sectioned into 70 nm ultrathin slices using an ultramicrotome. The sections were stained with 2% uranyl acetate in ethanol for 8 min in the dark, followed by 2.6% lead citrate staining for 8 min, also in the dark, to prevent CO₂ contamination. Finally, the sections were analyzed using a HITACHI HT7800 transmission electron microscope.

### 2.11 Cell immunofluorescence staining

Cell suspensions were plated in confocal culture dishes at a density of 1 × 10^4^ cells/mL. After 24 h of stable growth, the medium was replaced according to the conditions of the experimental groups. Calcium ions were labeled using the Fluo-4/AM (Oregon, USA, F14217) calcium fluorescent indicator on days 7, 14, and 21 of culture. Fluorescence images were captured with a laser confocal microscope (Olympus, Japan, CX31), and fluorescence intensity was quantified using ImageJ software.

### 2.12 Western blot (WB)

For protein analysis, cell suspensions were seeded into culture dishes at a density of 1 × 10^4^ cells/mL. After 24 h, the medium was replaced based on the experimental group conditions. Protein expression at days 7, 14, and 21 post-culture was assessed via WB. Total protein was extracted using RIPA lysis buffer containing protease inhibitors (Beijing Zoman Biotechnology Co., Ltd., ZS306-1), and protein concentration was measured using a BCA kit (Beijing Zoman Biotechnology Co., Ltd., ZS301-2). Equal amounts of protein (20 µg) were separated by 10% sodium dodecyl sulfate polyacrylamide gel electrophoresis (Biotides, Beijing, China) and transferred onto polyvinylidene fluoride membranes (Immobilon, USA, 3098530). Following blocking with 5% skimmed milk, the membranes were cut according to the molecular weight of the prestained marker protein and incubated overnight at 4°C with primary antibodies. The membranes were subsequently incubated with horseradish peroxidase-conjugated anti-rabbit or anti-mouse IgG secondary antibodies (1:5,000; Yeasen, China, 34854ES60). Protein bands were detected using an ECL reagent (Zoman, China, ZD310) and visualized with a 6100 EXP system (Clinx Science Instruments, Shanghai, China). Band intensities (gray values) were quantified with ImageJ software for comparative analysis. Primary antibodies included anti-Col I, anti-Homer1, anti-Runx2 (1:1000; Affinity, USA, AF5186), anti-AKT (1:5,000; Huabio, China, WL0003b), and anti-ACTB (β-actin; 1:50,000; Abclonal, China; AC026).

### 2.13 RNA extraction and quantitative real-time PCR

Total RNA was extracted from tissues and cells using the EaStep^®^ Super Total RNA extraction kit (Mei5 Biotechnology Co., Ltd., Beijing, China) as previously described in Section 1.12. RNA concentration and quality were assessed using a spectrophotometer. Reverse transcription was performed according to the manufacturer’s protocol (Mei5 Biotechnology, Beijing, China, MF167-01) to synthesize cDNA. qRT-PCR was conducted using the 2× M5 HiPer SYBR Premix EsTaq (Mei5 Biotechnology Co., Ltd., Beijing, China, MF787-T) and a QuantStudio™ Real-time PCR Detection System (Applied Biosystems). The PCR program consisted of an initial denaturation at 95°C for 30 s, followed by 40 cycles of 5 s at 95°C and 32 s at 60°C. Relative gene expression levels were calculated using the 2^‒ΔΔCt^ method, with GAPDH as the internal reference. Primer sequences for qRT-PCR are provided in Table 1.

**Table 1:** Nucleotide sequences of real-time PCR primers

Name	Primer sequence
CaSR	Forward: 5′-CTTTGTGCTCTGTATCTCGT-3′
	Reverse: 5′-TTGGCTTCAAATACCAGGAG-3′
Homer1	Forward: 5′-TGAGTGTTTTCCATGTCCAA-3′
	Reverse: 5′-CTGACAAGCTGGGACTTTAT-3′
Runx2	Forward: 5′-ACCAACCGAGTCATTTAAGG-3′
	Reverse: 5′-TCCCAAAAGAAGTTTTGCTG-3′
AKT	Forward: 5′-TCACAGATGCAGCTACCATGAAGAC-3′
	Reverse: 5′-AGGCAACCTCCCACACATCATTTC-3′
Col Ⅰ	Forward: 5′-CAAAGGTGCTACATCTCTGT-3′
	Reverse: 5′-CACACAAGTCCCTATCCATT-3′
GAPDH	Forward: 5′-AGTGCCAGCCTCGTCTCATA-3′
	Reverse: 5′-ATGAAGGGGTCGTTGATGGC-3′

### 2.14 Statistical analyses

Data are presented as mean ± standard deviation (SD) and analyzed using SPSS 20 software (SPSS Inc., Chicago, IL). Group comparisons were made using a *t*-test. For normally distributed data with homogeneity of variance, one-way ANOVA was used for multiple group comparisons, followed by the least significant difference test for pairwise comparisons. For non-normally distributed data, the rank-sum test was applied. For normally distributed data with unequal variances, Tamhane’s T2 test was used. A *P*-value <0.05 was considered statistically significant.

## 3 Results

### 3.1 Postmenopausal osteoporosis in rats leads to decreased expression of CaSR and Homer1

OP development in bilateral OVX rats was evaluated using micro-CT, three-point bending tests, and H&E staining.

**Figure 2:**
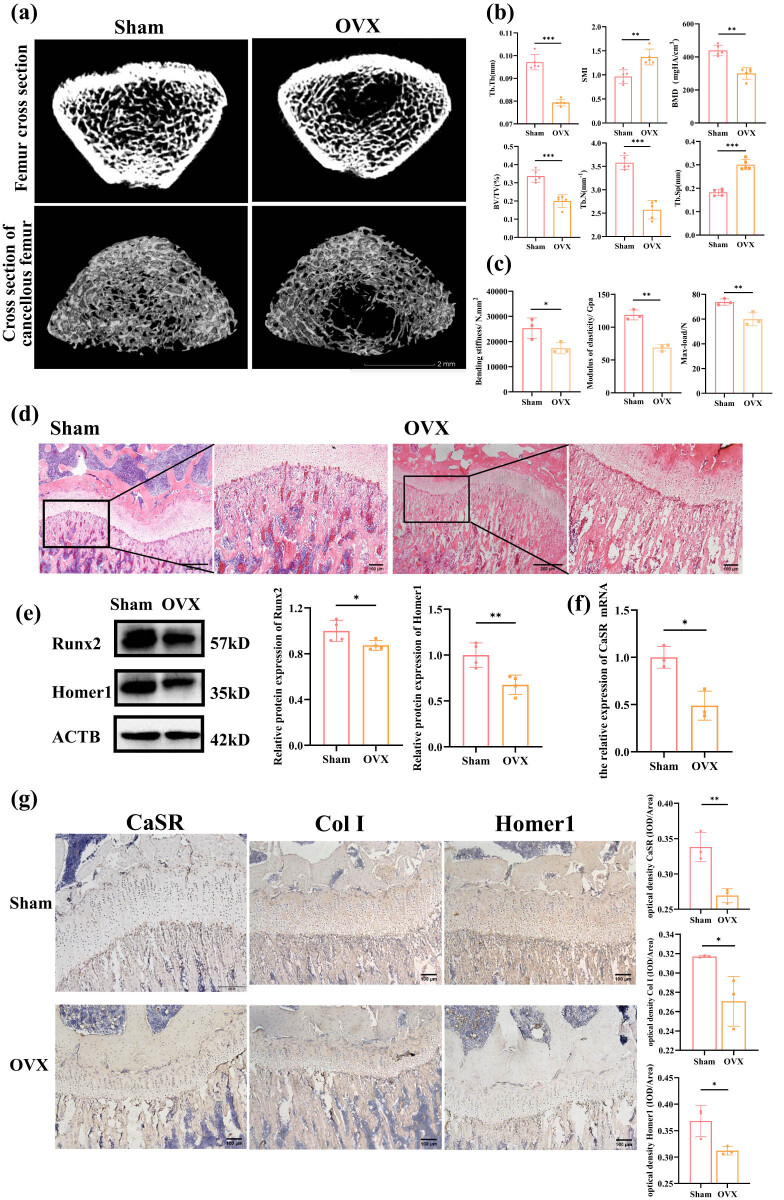
Expression of CaSR and Homer1 in OVX rats: (a) trabecular bone images of rats; (b) bone formation indices, including BMD, BV/TV, SMI, Tb.Th, Tb.N, and Tb.Sp as analyzed by micro-CT; (c) biomechanical parameters of rat tibia: maximum load, bending stiffness, and elastic modulus; (d) bone formation in rats assessed by H&E staining (40 ×, 100 ×); (e) CaSR mRNA expression; (f) Runx2 and Homer1 protein expression in femur tissues; and (g) IHC analysis of CaSR, Col I, and Homer1 expression in tibia. ^*^ *P* < 0.05; ^**^ *P* < 0.01.

Micro-CT analysis revealed significant differences between OVX and sham rats. Trabecular bone imaging indicated a marked reduction in bone mass in OVX rats compared to sham rats (*P* < 0.05, Figure 2a). Longitudinal changes in bone microarchitecture are summarized in Figure 2. OVX rats exhibited significant reductions in BMD, BV/TV, Tb.Th, and Tb.N, along with increases in SMI and Tb.Sp (*P* < 0.05, *n* = 3, Figure 2b). Biomechanical testing by three-point bending demonstrated significantly lower maximum load, bending stiffness, and elastic modulus in OVX rats relative to sham rats (*P* < 0.05, *n* = 3, Figure 2c). H&E staining of the proximal tibia revealed clear morphological differences between the groups. The growth plate in OVX rats appeared thinner, and the trabecular bone beneath it showed a markedly thinner reticular structure compared to the sham group (Figure 2d). These results confirm the development of OP in OVX rats, characterized by reduced bone mass, impaired biomechanical properties, and altered bone microarchitecture.

Additionally, WB and qPCR analyses showed significant reductions in both protein and mRNA expression levels in OVX rats compared to sham controls. The relative protein expression of Runx2 and Homer1 was significantly decreased in OVX rats (*n* = 3, *P* < 0.05, Figure 2e). Similarly, the relative mRNA expression of CaSR was notably lower in OVX rats compared to sham rats (*n* = 3, *P* < 0.05, Figure 2f).

Immunohistochemical analysis confirmed decreased expression and distribution of Col I, CaSR, and Homer1 proteins near the growth plate in OVX rats compared to sham rats (*P* < 0.05, Figure 2g).

These results suggest that Homer1 and CaSR are closely associated with the development of OP. Therefore, Homer1 and CaSR may modulate OP through the regulation of osteogenesis.

### 3.2 CaSR regulates the OB differentiation

**Figure 3:**
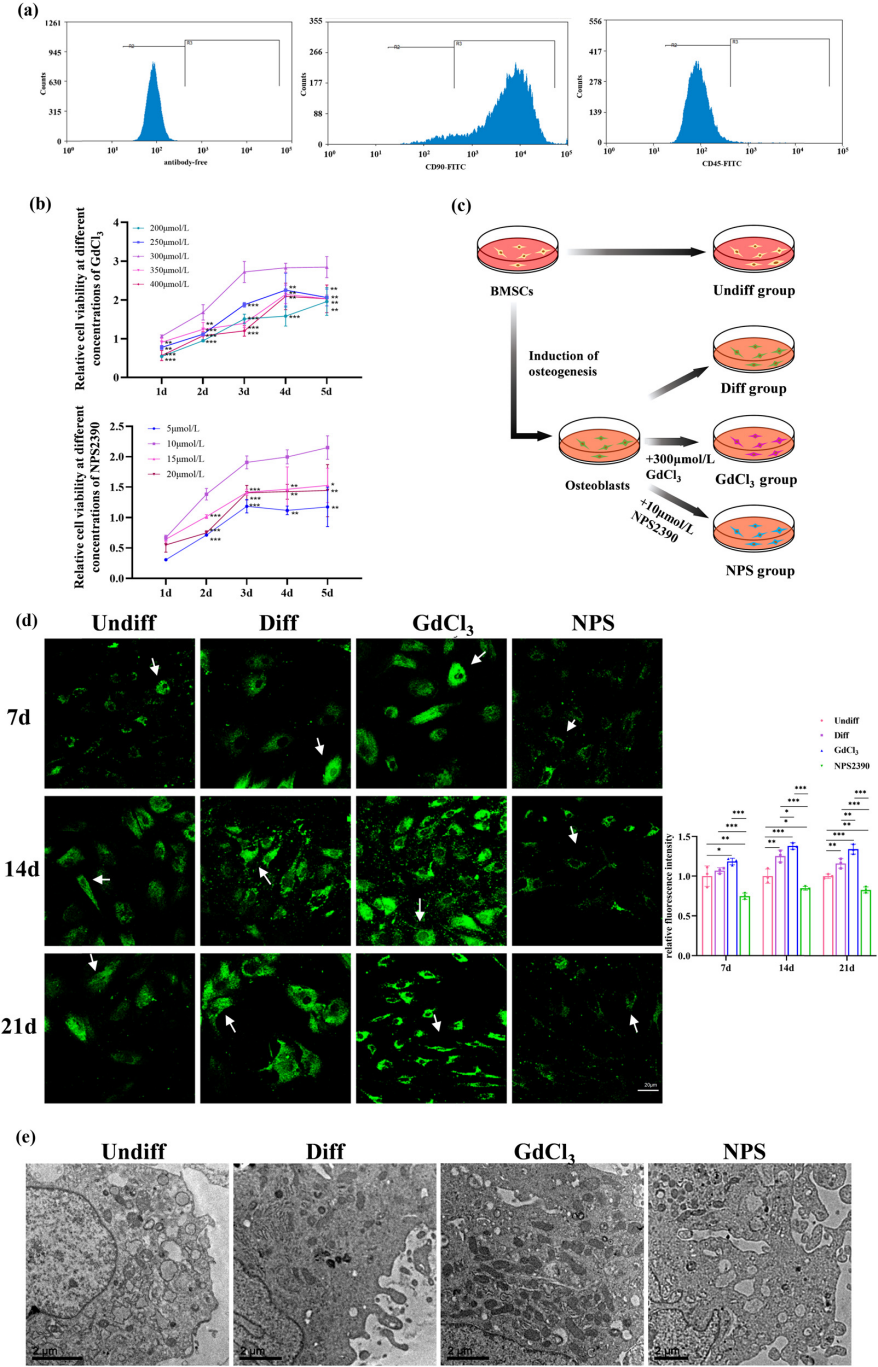
CaSR regulates the OB differentiation of BMSCs: (a) flow cytometry analysis for BMSC identification; (b) determination of optimal concentrations for GdCl_3_ and/or NPS 2390 based on cell proliferation; (c) experimental design schematic illustrating the impact of CaSR on BMSC osteogenic differentiation; (d) effect of CaSR on calcium distribution in BMSCs. Green fluorescence represents Fluo-4/AM calcium indicator staining in the cytoplasm, with arrows highlighting calcium-positive areas (×400 magnification); and (e) ultrastructural observation of cells after 14 days of osteogenic induction. ^*^ *P* < 0.05; ^**^ *P* < 0.01; ^***^ *P* < 0.001.

To investigate the role of CaSR in OB differentiation, BMSCs at passage 3 were first identified. Flow cytometry analysis showed a high purity of the BMSCs, with 93.29% positive expression for CD90 and 0.91% negative expression for CD45 (Figure 3a). Cells without antibodies served as the control group. The concentrations of the CaSR agonist (GdCl_3_) and inhibitor (NPS2390) were then optimized by evaluating cell viability through CCK-8 assays. BMSCs were treated with various concentrations of GdCl_3_ (200, 250, 300, 350, and 400 μmol/L) and NPS2390 (5, 10, 15, and 20 μmol/L). The results indicated that 300 μmol/L GdCl_3_ and 10 μmol/L NPS2390 were optimal for promoting cell proliferation (*P* < 0.05, *n* = 6, Figure 3b). These concentrations were used in subsequent experiments.

To validate the experimental setup, group experiments were conducted (Figure 3c). The activation or inhibition of CaSR was confirmed by assessing intracellular calcium levels in different experimental groups after induction.

The fluorescence intensity in NPS2390-treated cells was significantly lower than that in the other three groups on day 7 of induction (*P* < 0.05). Over time, changes in calcium ion fluorescence were observed. By days 14 and 21, fluorescence intensity in the GdCl_3_ group was notably stronger than in the Diff group, while the NPS group exhibited weaker fluorescence. These results suggest that calcium ion pathways can be modulated by altering CaSR expression, confirming the successful activation and inhibition of CaSR in the experimental setup (*P* < 0.05, *n* = 3, Figure 3d).

Further examination of intracellular organelles using transmission electron microscopy revealed distinct alterations between experimental groups. In the Undiff group, BMSCs exhibited typical mesenchymal cell characteristics, including short microvilli, abundant rough endoplasmic reticulum, and intact mitochondria. After 14 days of induction, noticeable changes were observed. In the Diff group, BMSCs exhibited increased microvilli, irregular nuclear morphology, and significant glycogen accumulation in the cytoplasm. Prominent organelles such as ribosomes, rough endoplasmic reticulum, and Golgi apparatus suggested OB-like differentiation. In the GdCl_3_ group, BMSCs showed normal mitochondrial structure, with a significant increase in mitochondrial numbers compared to the Undiff group. In contrast, the NPS2390 group displayed a reduction in mitochondrial numbers and evident structural abnormalities, such as cristae breakage, dissolution, and vacuolation (Figure 3e). These results confirm that CaSR modulation influences osteogenic differentiation.

### 3.3 Effect of CaSR on osteogenic differentiation of BMSCs

To elucidate the role of CaSR in osteogenic differentiation, the effects of CaSR activation and inhibition on the expression of key osteogenic markers, including Homer1, AKT, Runx2, and Col I, were assessed at both protein and mRNA levels.

**Figure 4:**
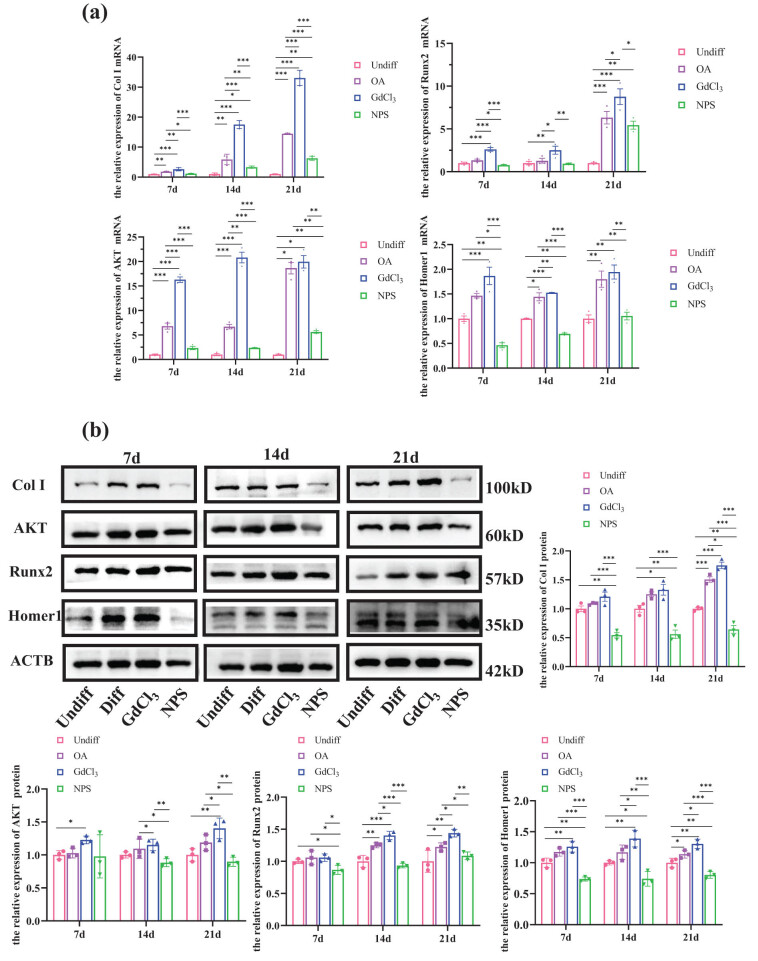
Effect of CaSR on osteogenic differentiation of BMSCs: (a) Reverse transcription quantitative real-time polymerase chain reaction analysis of mRNA expression for Col I, AKT, Homer1, and Runx2 in the four groups and (b) protein expression of Col I, AKT, Runx2, and Homer1 detected by WB. *P < 0.05; **P < 0.01; ***P < 0.001.

The results indicated that, compared to the Diff group, the relative protein expression and mRNA levels of these markers were significantly increased with the addition of the CaSR agonist (GdCl_3_) and significantly decreased with CaSR inhibition (NPS2390) (*P* < 0.05, *n* = 6, Figure 4a and b). These results suggest that CaSR activation enhances the osteogenic differentiation of BMSCs by promoting the expression of AKT, Homer1, Runx2, and Col I.

## 4 Discussion

This research indicates that the expression levels of CaSR and Homer1 were significantly lower in the OVX group compared to the Sham group, which may contribute to the impaired osteogenic differentiation capacity observed in the OVX rats. OP accelerates the aging of ovariectomized stem cells, thereby diminishing their osteogenic potential. Previous studies have suggested that BMSCs may be negatively affected in OP, which could influence the progression of the disease [18]. Consequently, understanding the mechanisms underlying the osteogenic differentiation of BMSCs is critical for advancing our knowledge of OP pathogenesis.

BMD is a widely accepted metric for assessing bone loss, while BV/TV serves as a reliable indicator of trabecular and cortical bone mass. Parameters such as Tb.Sp and Tb.N are primarily used to evaluate the spatial structure of trabeculae, and SMI and Tb.Sp are commonly employed to assess bone compressive strength [19]. Runx2 and Col I are the well-established markers of osteogenesis [20]. Osteoporotic animal models are invaluable for studying bone formation and remodeling processes [15]. In this study, an osteoporotic rat model (OVX model) was successfully established through ovariectomy. The OVX rats exhibited significant reductions in BMD, BV/TV, Tb.N, and Tb.Th, along with marked increases in SMI and Tb.Sp. The trabecular bone structure was sparse and defective, with evident microstructural damage, leading to a substantial reduction in bone strength. Additionally, the expression of Runx2 and Col I proteins in femoral tissue was significantly downregulated. These results align with previous studies on osteoporosis, confirming that the ovariectomy procedure effectively replicated a postmenopausal osteoporosis (PMOP) rat model [21].

Significant downregulation of CaSR expression was observed in the femoral tissue and growth plate region of OVX rats, suggesting that CaSR may play a key regulatory role in the physiological changes in these animals. As a GPCR, CaSR is critical for osteogenic differentiation and bone repair in BMSCs [22]. Gadolinium chloride (GdCl_3_), a potent activator of CaSR [23], and NPS2390, a highly selective antagonist of Group I metabolic glutamate receptors (mGluRs), share high homology with CaSR and mGluR1, making NPS2390 a widely used CaSR antagonist [7,24]. In this study, Ca^2+^ expression increased upon CaSR activation and decreased with its inhibition, confirming the successful modulation of CaSR activity. Ultrastructural observations via transmission electron microscopy revealed abundant mitochondria following CaSR activation, suggesting enhanced energy availability, which may directly support OB function. Col I, a key marker of early osteogenic differentiation, is essential for determining the mechanical and elastic properties of bone tissue [25]. Similarly, Runx2, known as a master regulator of bone development and OB differentiation, promotes the commitment of mesenchymal stem cells to the OB lineage [26]. Runx2 activation regulates Col I gene expression, facilitating successful OB differentiation [27].

CaSR activation significantly elevated serum levels of Runx2 and Col I in OVX rats, while the inhibition of CaSR reduced these levels. This suggests that CaSR activation regulates Runx2 gene expression, promoting Col I synthesis, thereby maintaining extracellular matrix homeostasis and promoting osteogenic differentiation of BMSCs. These observations align with previous studies, such as those by Xu et al. [28], which demonstrated that CaSR enhances BMSC proliferation, and An [29], who reported that CaSR activation induces both vasodilation and osteogenic differentiation.

Furthermore, CaSR has been shown to promote osteogenic differentiation of Wharton’s jelly mesenchymal stem cells by modulating the PI3K/AKT signaling pathway [30]. CaSR also influences AKT expression in various contexts, including osteosarcoma cell proliferation [31], human telomerase reverse transcriptase in gastric cancer [32], and neurite outgrowth in developing chicken embryos [33]. The PI3K/AKT pathway plays a pivotal role in numerous cellular processes, including metabolism, growth, and proliferation. Upon activation by protein kinases and GPCRs, PI3K converts PIP2 to PIP3, creating high-affinity binding sites for the PH domain of AKT, which promotes AKT translocation to the membrane. There, phosphorylation at Ser473 and Thr308 activates downstream transcription factors. The PI3K/AKT pathway is also a central regulator of OB differentiation, facilitating OB differentiation and mineralization by upregulating osteogenic transcription factors such as Runx2 and Osterix, as well as extracellular matrix proteins like Col I while enhancing ALP activity. Additionally, traditional Chinese medicine compounds, such as myricetin, have been shown to enhance osteogenic differentiation of immortalized bone marrow mesenchymal stem cells and reduce bone loss in OVX mice via the PI3K/AKT pathway. However, the specific involvement of AKT in the mechanisms observed in this study remains to be determined.

The HOMER1 gene, which encodes the HOMER1 protein, is a critical scaffold involved in signal transduction and various neurodevelopmental processes, including chronic pain and drug addiction [34,35]. In OBs, studies have established a link between CaSR, HOMER1, and AKT, where CaSR activation induces AKT phosphorylation at Ser473 and facilitates the nuclear translocation of β-catenin [36]. Additionally, membrane-bound HOMER1 proteins are known to promote AKT phosphorylation [37]. CaSR-mediated phosphorylation of AKT at Ser473, subsequent phosphorylation of GSK3β, and the nuclear translocation of β-catenin are closely associated with HOMER1. In MG63 osteosarcoma cells, CaSR and HOMER1 co-regulate AKT Ser473 and GSK3β-S9 phosphorylation. Similarly, studies in human OBs have identified a protein complex consisting of CaSR, HOMER1, and mTORC2 that governs the AKT phosphorylation pathway. This interaction significantly enhances OB proliferation and differentiation [13]. These findings emphasize the pivotal role of HOMER1 in integrating CaSR and AKT signaling during osteogenesis. By acting as a key mediator, HOMER1 facilitates bone remodeling and OB differentiation, positioning it as a potential therapeutic target for promoting bone formation and repair.

In this study, CaSR upregulation led to a significant increase in Homer1 and AKT protein levels, while CaSR inhibition resulted in a marked reduction in these proteins. This suggests that, during osteogenic differentiation, CaSR and Homer1 are co-regulated, with CaSR acting as a positive regulator that enhances Homer1 expression and promotes AKT activation.

In conclusion, an OVX rat model was established, revealing reduced CaSR and Homer1 expression in OVX rats. *In vitro* experiments further examined the roles and mechanisms of CaSR and Homer1 in osteogenic differentiation of BMSCs. However, this study has several limitations. First, while the activity of Homer1 and AKT was confirmed following CaSR modulation *in vitro*, the precise mechanisms underlying CaSR regulation remain incompletely understood. Second, the role of Homer1 in these regulatory pathways requires further validation. Finally, whether CaSR can mitigate OP by modulating Homer1 *in vivo* remains to be explored. Future research should address these limitations by investigating the underlying mechanisms of CaSR regulation and further validating the involvement of Homer1. These efforts will provide deeper insights into targeting CaSR and Homer1 as potential therapeutic strategies for OP.

## 5 Conclusions

In summary, this study demonstrates the expression of CaSR in the bone tissue of SD rats and highlights the potential involvement of CaSR and Homer1 pathways in OB differentiation. CaSR effectively promotes osteogenic cell differentiation, likely through Homer1-mediated regulation of AKT phosphorylation, which in turn facilitates BMSC differentiation. These findings suggest that modulating CaSR activity may offer a promising therapeutic strategy for bone diseases. Future research should focus on investigating various CaSR activators and inhibitors, as well as their underlying mechanisms in bone tissue under both physiological and pathological conditions, to enhance our understanding and inform potential therapeutic applications.
